# Validation of Death and Dying Distress Scale-Chinese Version and Prevalence of Death Anxiety Among Patients With Advanced Cancer

**DOI:** 10.3389/fpsyt.2021.715756

**Published:** 2021-10-20

**Authors:** Lili Tang, Yening Zhang, Ying Pang, Yuhe Zhou, Jinjiang Li, Lili Song, Yi He, Zimeng Li, Yan Wang

**Affiliations:** Department of Psycho-Oncology, Key Laboratory of Carcinogenesis and Translational Research (Ministry of Education/Beijing), Peking University Cancer Hospital and Institute, Beijing, China

**Keywords:** oncology, death anxiety, death and dying distress scale, advanced cancer patients, validation, palliative care, psychology, psychometrics

## Abstract

**Purpose:** Death anxiety is commonly experienced by individuals with advanced cancer who have a limited life expectancy. The Death and Dying Distress Scale (DADDS) is a validated measure that was created to capture this experience; but no Chinese version is available to date. We conducted a cross-sectional study to explore the psychometric properties of a Chinese version DADDS (DADDS-C) and address prevalence of death anxiety among patients with advanced cancer.

**Methods:** Patients with advanced cancer were recruited from Peking University Cancer Hospital. Measures administered included: DADDS-C, Patient Health Questionnaire (PHQ-9), General Anxiety Disorder-7(GAD-7), Quality of Life at End of Life in Cancer (QUAL-EC), Functional Assessment of Chronic Illness Therapy-Spiritual Well-Being Scale (FACIT-sp). McDonald's Omega, Cronbach's alpha, Exploratory Factor Analysis and Confirmatory Factor Analysis were used to test DADDS-C's reliability and validity. Logistic regression analysis was used to identify risk factors for death anxiety.

**Results:** Of 300 patients approached, 256 (85%) provided informed consent and completed the questionnaires. Of these participants, 43 (16.8%) had moderate death anxiety based on scores of ≥45 on the DADDS-C. Three factors (feeling shortness of time, dying and death distress, being a burden to others) explained 71.643% of shared variation with factor loadings ranging from 0.629 to 0.822. Cronbach's alpha was 0.939; Omega total was 0.959. DADDS-C had acceptable convergent and discriminant validity. Logistic regression analysis indicated that two factors (better relationship with healthcare providers and preparation for end of life) protected patients from death anxiety.

**Conclusion:** DADDS-C is a valid tool for measuring death anxiety in Chinese patients with advanced cancer. The presence of at least moderate death anxiety in a substantial minority of these patients calls for screening for this symptom and for more routine psychological interventions to alleviate and prevent such distress in this population.

## Introduction

Cancer is still one of the leading causes of death in China, with almost four million new cancer cases in 2015 and more than 2 million cancer deaths (1). Although the 5-year survival rate for of all kinds of cancer in the world has improved in recent years, that for most cancer types in China is still lower than in developed countries (2, 3). Further, the symptom burden of those living with advanced cancer is substantial (4–6), with moderate to severe symptoms of depression and demoralization reported in almost one quarter of such individuals (7, 8) and more than 40% reporting moderate to severe symptoms of death anxiety (9). Sussman had defined death anxiety as ‘psychological distress in the form of anxiety about one’s death that contributes to functional impairment in one's life' (10). Though some of patients with death anxiety didn't meet all criteria of the Diagnostic and Statistical Manual of Mental Disorders (DSM)-anxiety disorder, this group of patients might have poorer death quality (11). Terror management theory (TMT) suggested that death anxiety is a fundamental human fear that is amplified by mortality salience (awareness that one's death is inevitable), different from general anxiety which is triggered by excessive worry about practical problems in daily life (12). Based on this theory, self-esteem (experiencing sense of meaning and value) was the core protective factor for death anxiety and was suggested to be integrated into death anxiety management (13).

Talking about death and dying is taboo in many cultural systems, which makes it difficult for it to be openly discussed and studied in many settings (14, 15). It is common in this circumstance to see patients, families and medical staff be aware of the imminence of death and dying but to never discuss it openly (16). This taboo on open discussion among Chinese people is evident in the Confucian concept of ‘highlighting birth and avoiding death’ (17). Confucian ideology have deeply influenced Chinese culture, which is a background for Chinese patients' outlook on life. Benevolence, righteousness, courtesy, wisdom and trust are five core ideas, which has resulted in respecting the elderly and caring more for family development than individuals, as said by Confucius: to subdue Oneself and Return to the Proprieties is Perfect Virtue (18). This ideology also has influenced individuals' thinking about death and dying.

Death anxiety has been shown to be common in patients with advanced cancer and associated with other psychological disturbances. At least moderate death anxiety has been reported by 32% of a mixed sample of patients with advanced cancer (19) and in 43% of patients with non-small cell metastatic lung cancer (9). Death anxiety in these and other studies has been associated with depression, demoralization, fear of disease progression and with less attachment security (9, 20–24).

Some measures of death anxiety have been validated in Chinese (25, 26), but none has been specifically designed for patients with advanced cancer. Yang reported on the use of a Chinese version of Templer's Death Anxiety Scale (DAS) for colorectal cancer patients, but no other studies using this tool in China have been reported. Some items on the DAS are not tailored for individuals with advanced cancer, such as item 11 “I am really scared of having a heart attack,” and item 13 “I shudder when I hear people talking about a world war III” (26).

The Death and Dying Distress Scale (DADDS) was developed and validated by a clinical research team at Princess Margaret Cancer Center in Toronto, to assess distress about dying and death in individuals with advanced cancer (27, 28) and has been validated in German (21). The advantages for this scale were as below: (1) it was designed specifically for cancer patients and practical for further interventional researches among this population; (2) all items focused on death and dying distress and no specific scenarios, which was beneficial for generalizing utilization among patients with limited life. Recent research demonstrated that the DADDS has two subfactors, which are Finitude, referring to distress about the perceived shortness of time, and Dying, which refers to distress about the process of dying and death (29).

The present cross-sectional study is a preliminary examination of the validity of the Death and Dying Distress Scale-Chinese version (DADDS-C) in patients with advanced cancer. It was conducted in preparation for a randomized controlled trial (RCT) of Managing Cancer and Living Meaningfully (CALM) therapy in Chinese patients with advanced cancer “(ID: ChisCRT1900023129).” We hypothesized that DADDS-C would be a valid measurement for death anxiety with two factors as the former researchers suggested (item 1–10 for factor 1-Finitude, item 11–15 for factor 2-Dying) (29), could be used for death anxiety assessment, and death anxiety would be positively associated with anxiety, depression, lower quality of life and negatively associated with spiritual well-being. This cross-sectional study was designed to: (1) test the structure and validation of DADDS-C locally with factor analyses; (2) addressing psychometric priority about this measurement; (3) explore the prevalence and independent risk factors of death anxiety among Chinese patients with advanced cancer through multiple regression analysis.

## Materials and Methods

### Participants and Procedure

This cross-sectional study was approved by Peking University Cancer Hospital Ethics Committee (No. 2018YJZ24) on 25 May, 2018. Participants were a convenience sample of inpatients and outpatients with advanced cancer recruited from Peking University Cancer Hospital from June, 2018 to December, 2018, and the patients were approached both from oncology wards and psycho-oncology clinic. We trained study coordinators and checked their competence of evaluation consistency for delivering all measurements in this study. Inclusion criteria for participants were: (1) age ≥18 years old; (2) pathology diagnosis of advanced cancer [Union for International Cancer Control-UICC TNM classification of Malignant Tumor, stage III and stage IV (30)]; (3) expected survival time ≥6 months; (4) being able to provide informed consent; (5) education level ≥ primary school or the ability to understand the questionnaires. Patients with moderate to severe cognitive dysfunction (according to psychiatrists' interview before registered) and those receiving psychosocial intervention or anti-psychotic treatments were excluded. Most participants answered questionnaires fluently by themselves; some of them were assisted by study coordinators. All 256 participants were attached for repeat assessment by phone 7–14 day after the initial completion, but only 27 patients finished the re-test; no demographic difference was found between this 27 group and the other patients who rejected re-test. The re-test was rejected mostly by patients' family members, because they thought the items in DADDS-C would trigger patients' negative emotion. Sufficient data were therefore not available to determine test-retest reliability.

### Translation of DADDS-C

We completed the translation according to the 5 steps of World Health Organization (WHO) translation methodology (31). In collaboration with the developers of the DADDS at the Princess Margaret Cancer Center, a bilingual team of experienced psychologists and psychiatrists (more than 3-years' experience of being an interpreter and translator on psycho-oncology) translated it into simplified Chinese, which was the authority language and commonly used by majority population in Mainland China. One oncologist professor with language proficiency only in Chinese completed the first translated simplified Chinese version and made some comments. The bilingual team considered these comments and revised the documents, then back-translated into English. The accuracy of the back-translation was confirmed with the team at the Princess Margaret Cancer Center. We conducted pilot testing of DADDS-C with 10 outpatients and their family members; one patient's family member refused to allow the patient to complete the questionnaire, and the other 9 patients and family members provided ratings for all items. No negative feedback was provided by them.

### Measures

Demographic and clinical information included: age, sex, religion, marital status, education level, residential status, average family income, medical insurance coverage, diagnosis, and ongoing therapies.

The DADDS is a 15 item self-report scale of distress about death and dying, which has been validated in both English and in German (21, 29). Items are rated on a 6-point Likert scale (0 = I did not experience this thought or concern; 1 = I experienced very little distress; 2 = I experience mild distress; 3 = I experienced moderate distress; 4 = I experienced great distress; 5 = I experienced extreme distress). We retained all 15 items and each item was rated on 6-point Likert scales in DADDS-C. Total scores ranged from 0 to 75. As suggested by Neel (19), a cutoff point of 45 was used to define death anxiety as a dichotomous variable (0 = none to mild distress; 1 = moderate to extreme distress).

The Patient Health Questionnaire (PHQ-9) is a 9-item scale for assessing depression among patients, with total score ranges from 0 to 27. It has been validated in Chinese population, with the Cronbach's is 0.89 and the cutoff point of ≥10 was used to define moderate depression (32).

Anxiety was assessed by General Anxiety Disorder-7 (GAD-7), a widely used self-reported scale for anxiety, which has been validated in Chinese general hospital outpatients, with the Cronbach's is 0.898 (33). Total scores range from 0 to 27, with ≥10 used as the cutoff for clinical cases of anxiety (33).

Quality of Life at the End of Life-Cancer (QUAL-EC) validated by Lo et al. (34), is a short version of QUAL-E developed by Steinhauser et al. (35). QUAL-EC contains 17 items (1–5 point scale), with subscales of: (1) symptom burden (range score 3–15), with lower score reflecting better outcome; (2) relationship with healthcare provider (range score 5–25), with higher score reflecting better outcome; (3) preparation for end-of-life (range score 4–20), reverse-scored, with lower scores reflecting better outcome; (4) life completion (range score 5–25), with higher scores reflecting better outcome. Subscale scores were used for statistical analysis. The Cronbach's for the subscales were: 0.83 for Symptom Burden, 0.73 for preparation for End-of-Life, 0.83 for Life Completion and 0.80 for Relationship with Healthcare Provider. We translated the QUAL-EC into simplified Chinese by the same translation method as described above for the DADDS-C.

We obtained the simplified Chinese version of the Functional Assessment of Chronic Illness Therapy-Spiritual Well-being (FACIT-sp) from the official website (36) and received a license to use it. This questionnaire has been validated in Chinese cancer patients, with the Cronbach's for subscales ranges from 0.711 to 0.920 (37). This simplified Chinese version of FACIT-sp has 12 items, 5-point Likert scale for items (0–4), two items (4 and 8) should be reverse-scored. It contains two subscales: meaning/peace and faith. Total score was calculated based on the official FACIT-Sp scoring instruction.

### Statistical Analysis

Data from questionnaires with more than half of the items missing were not included in the analyses. The demographic and medical information were summarized descriptively in Table 1. One-way ANOVA (if homogeneity of variance assumption was satisfied) and non-parametric Kruskal-Wallis H test (if homogeneity of variance assumption was not satisfied) were used to compare DADDS-C scores among demographic and medical characteristics. The results determined which demographic and medical characteristics would be fully considered to enter the logistics regression.

**Table 1:** Social demographic and medical information, discrepancy of DADDS among different groups.

**Subject**	**N (%)/M ± SD**	**DADDS**,	**χ^2^ or ***F***-value**	***P*** **-value**
	**(***N*** = 256)**	**M ± SD**		
Age (y)	50.73 ± 11.35		χ^2^ = 28.859	<0.001**
≤ 44	76 (29.7)	31.59 ± 21.962		
45–59	118 (46.1)	20.77 ± 17.325		
≥60	62 (24.2)	12.69 ± 10.826		
Sex			*F* = 0.591	0.443
Male	30 (11.7)	19.539 ± 17.544		
Female	226 (88.3)	22.369 ± 19.072		
Religious			*F* = 0.749	0.388
Yes	43 (16.8)	24.30 ± 20.085		
No	213 (83.2)	21.57 ± 18.654		
Marital status			*F* = 0.198	0.657
Without partner (single, separated, divorced, widowed)	26 (10.2)	20.46 ± 16.258		
With spouse	230 (89.8)	22.20 ± 19.187		
Education Level			χ^2^ = 14.456	0.006**
Primary school and lower	23 (9.0)	10.87 ± 10.047		
Junior middle school	62 (24.2)	21.23 ± 18.104		
High middle school	66 (25.8)	22.52 ± 19.480		
Junior college	41 (16.0)	19.93 ± 17.893		
Undergraduate and above	64 (25.0)	27.66 ± 20.381		
Residential			χ^2^ = 2.340	0.126
City	213 (83.2)			
Village	42 (16.4)			
Null	1 (0.4)			
Average family income			*F* = 1.877	0.155
<3,000 Yuan/month	53 (20.7)	20.83 ± 18.903		
3,000–5,000 Yuan/month	90 (35.2)	19.71 ± 17.797		
>5,000 Yuan/month	112 (43.8)	24.65 ± 19.543		
Null	1 (0.4)			
Medical coverage			*F* = 3.247	0.073
Public expense/insurance	91 (35.5)	24.88 ± 19.580		
Self-pay	165 (64.5)	20.45 ± 18.368		
Diagnosis			*F* = 0.401	0.753
Breast	186 (72.7)	21.26 ± 18.259		
Lung	20 (7.8)	24.65 ± 21.313		
Gastrointestinal	26 (10.2)	24.50 ± 19.014		
Others	24 (9.4)	23.08 ± 18.889		
Therapies (surgery, chemotherapy, radiotherapy)			χ^2^ = 4.316	0.229
None	44 (17.2)	21.18 ± 20.676		
Surgery, surgery and chemotherapy, surgery and radiotherapy	63 (24.6)	18.46 ± 15.583		
Chemotherapy, radiotherapy, chemotherapy and radiotherapy	57 (22.3)	26.37 ± 21.690		
Surgery and chemotherapy and radiotherapy	92 (35.9)	22.18 ± 18.889		
DADDS-C	22.03 (18.889)			
DADDS-C <45	43 (16.8)			
DADDS-C ≥ 45	213 (83.2)			
PHQ-9	7.13 (5.510)			
PHQ-9 <10	189 (73.8)			
PHQ-9 ≥ 10	67 (26.2)			
Suicide Ideation	64 (25.0)			
GAD-7	4.64 (4.953)			
GAD-7 <10	211 (82.4)			
GAD-7 ≥ 10	45 (17.6)			
FACIT-sp	25.95 (8.445)			
QUAL-EC symptom burden	10.07 (3.204)			
QUAL-EC relationship with health care provider	19.90 (4.078)			
QUAL-EC preparation for end-of-life	12.86 (4.061)			
QUAL-EC life completion	20.75 (4.375)			

** *p < 0.01*.

Validation test: Exploratory factor analysis (EFA) (Principal Component Analysis for extraction; Eigenvalue >1) was used to explore the underlying factors for DADDS-C using one-half sample (*N* = 128); Kaiser-Meyer-Olkin Measure and Bartlett's test were used to verify whether EFA could well-conducted. Three methods including Scree Plot (SP) (Eigenvalues >1), Parallel Analysis (PA), and Minimum Average Partial (MAP) were used to corroborate factors number (38). Confirmatory factor analysis (CFA) was used to test which one in two models of DADDS-C (one model was derived from EFA, the other model was suggestion by former research) was preferred using the other half sample (*N* = 128); the cut-off values of CFA indexes used to evaluate model fit were as follows (39): Chi-square/df ration (χ2/df) ≤ 2.0, standardized root mean square residual (SRMR) < 0.08, root mean square error of approximation (RMSEA) ≤ 0.06, comparative fit index (CFI) ≥0.95, Tucker-Lewis index (TLI) ≥0.95. Cronbach's alpha and McDonald's omega were used for internal consistency reliability. Spearman correlation test was used to explore relationship between DADDS-C total score with other variables (two tails test, with *p* < 0.05) to corroborate the criterion validity of DADDS-C.

Prevalence of death anxiety: descriptive analysis and binary logistic regression (Univariate and Multivariate Regression) were used to explore prevalence and significant risk factors for death anxiety.

Most data analyses were performed using SPSS 25.0 (IBM Corporation), except that McDonald's omega was conducted by R Studio (version 1.3.1093) and CFA was conducted by the SPSSAU project (2020) [Online Application Software], retrieved from http://www.spssau.com.

## Results

### Demographic and Medical Information

Three hundred patients approached by the study coordinators completed questionnaires. Two hundred and fifty-six valid data (i.e., rating of > 50% of items of all measures) entered into the database for analysis (85.3% validity ratio). The mean age of participants was 50.73 ± 11.35, with most being female, with breast cancer, having no religion, living in cities with spouse, having education level of high middle school and above, and paying for medical costs by themselves (Table 1).

### Descriptive Statistical Results of Questionnaires

The mean scores of the DADDS-C, PHQ-9, GAD-7, FACIT-sp, four subscales of QUAL-EC and the percentage of patients with moderate and severe death anxiety, depression, general anxiety, suicide ideation are all listed in Table 1. The Skewness and Kurtosis of DADDS-C were 0.922 and −0.063. The 6 most frequent symptoms reported on the QUAL-EC in descending order were: pain, fatigue, insomnia, nausea and vomiting, shortness of breath, and anxiety. The three most frequent items reported as the cause of distress on DADDS-C in descending order were: being a burden to others (2.78 ± 1.945), the impact of my death on my loved ones (2.60 ± 1.934), dying and death happening with a lot of pain or suffering (2.27 ± 2.018). Results of One-way ANOVA or non-parametric Kruskal-Wallis *H* test showed that the mean DADDS-C score differed by age and educational level but not by cancer type and sex (Table 1).

### Psychometric Properties of DADDS-C

#### Exploratory Factor Analysis (EFA) for DADDS-C

EFA using SP resulted in three factors which accounted 72.437% of variance in DADDS. Factor 1, accounting 32.124% variance in DADDS-C, consisted of items 1–7 about the shortness of time; Factor 2, accounting 29.534% of the variance in DADDS-C, consisted of items 10–15 that were about distress about dying and death; Factor 3, accounting 10.778% variance in DADDS-C, consisted of items 8 and 9, which were about the sense of being a burden to others, which was different from two factors reported by Shapiro (29) and one factor reported by Engelmann (21). High factor loadings (Table 2), ranged from 0.615 to 0.865. The 3 items with the highest factor loadings in each factor were: item 12 (in factor 2)-Be prolonged or drawn out (0.865); item 8 (in factor 3)-Being a burden to others (0.855); item 2 (in factor 1)-Not having said all that I want to say to the people I care about (0.811), which were helpful for defining factors names. Cronbach's alpha for the three factors were: factor 1–0.925, factor 2–0.659, and factor 3–0.898. PA suggested one factors being extracted and MAP suggested two factors. As one factor-model accounted only 38.898% of variance and two factors-model accounted 65.684%. We rejected the one factor-model and compared two factors-model and three factors-model using CFA below.

**Table 2:** Exploratory factor analysis of DADDS-C by Scree Plot (Eigenvalue > 1).

**Item**	**% of Variance**	**Cumulative %**	**Loading of factor 1**	**Loading of factor 2**	**Loading of factor 3**
1. Not having done all the things that I wanted to do	32.124%	32.124%	0.805	0.156	0.274
2. Not having said all that I want to say to the people I care about			0.811	0.248	0.176
3. Not having achieved my life goals and ambitions			0.799	0.301	0.114
4. Not knowing what happens near the end of life			0.697	0.395	0.148
5. Not having a future			0.645	0.521	0.123
6. The missed opportunities in my life			0.615	0.329	0.249
7. Running out of time			0.674	0.573	0.086
10. My own death and dying	29.534%	61.657%	0.473	0.649	0.150
11. Happen suddenly or unexpectedly			0.501	0.672	0.080
12. Be prolonged or drawn out			0.189	0.865	0.139
13. Happen when I am alone			0.338	0.747	0.005
14. Happen with a lot of pain or suffering			0.140	0.829	0.290
15. Happen very soon			0.553	0.700	0.042
8. Being a burden to others	10.778%	72.437%	0.090	0.157	0.855
9. The impact of my death on my loved ones			0.427	0.092	0.726

*The Kaiser-Meyer-Olkin Measure of Sampling Adequacy was 0.919, and Bartlett's test was significant (Chi square = 1461.561, p = 0)*.

#### Validity

**Figure 1:**
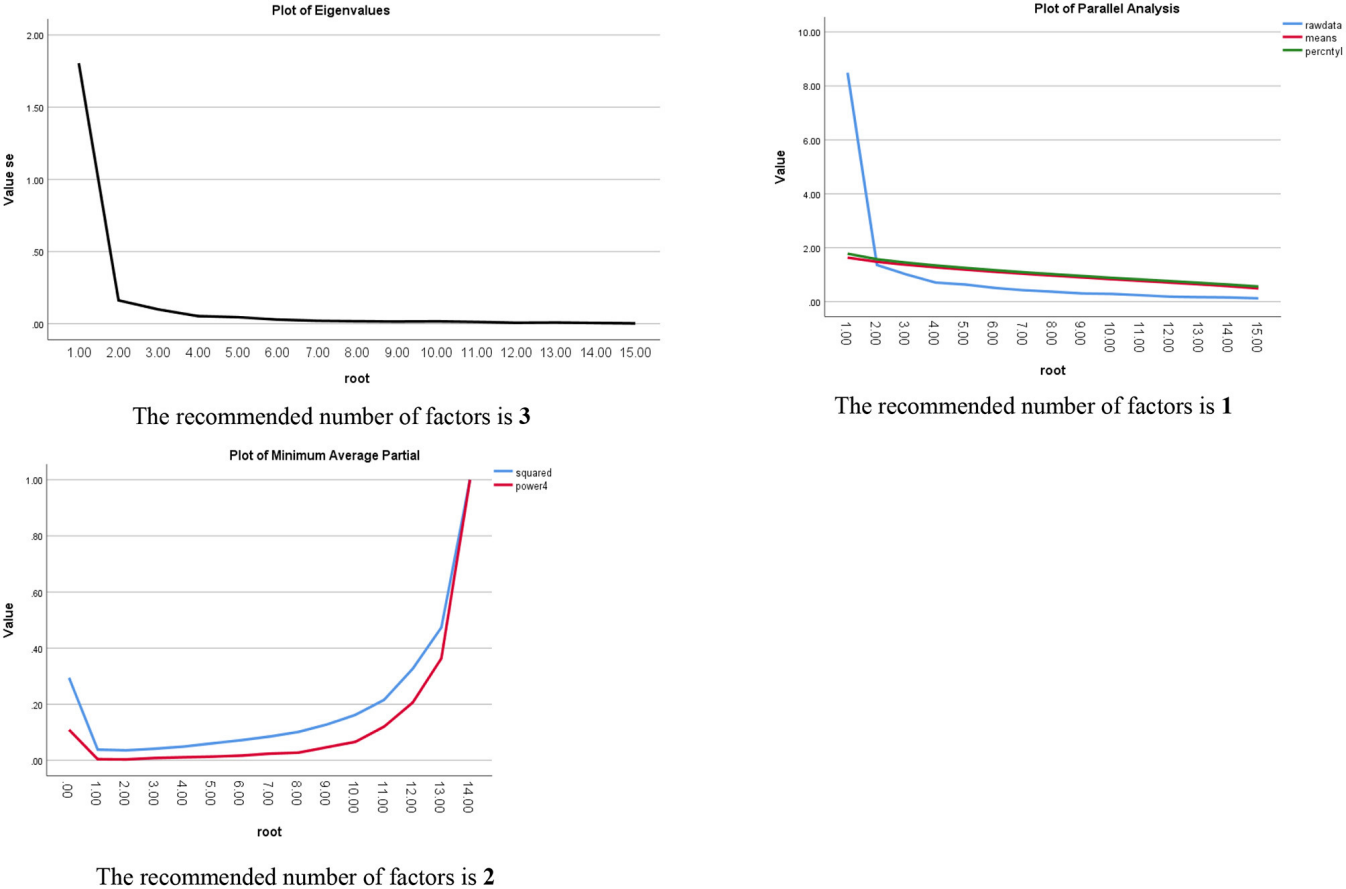
Plots of three methods to define factors numbers.

**Figure 2:**
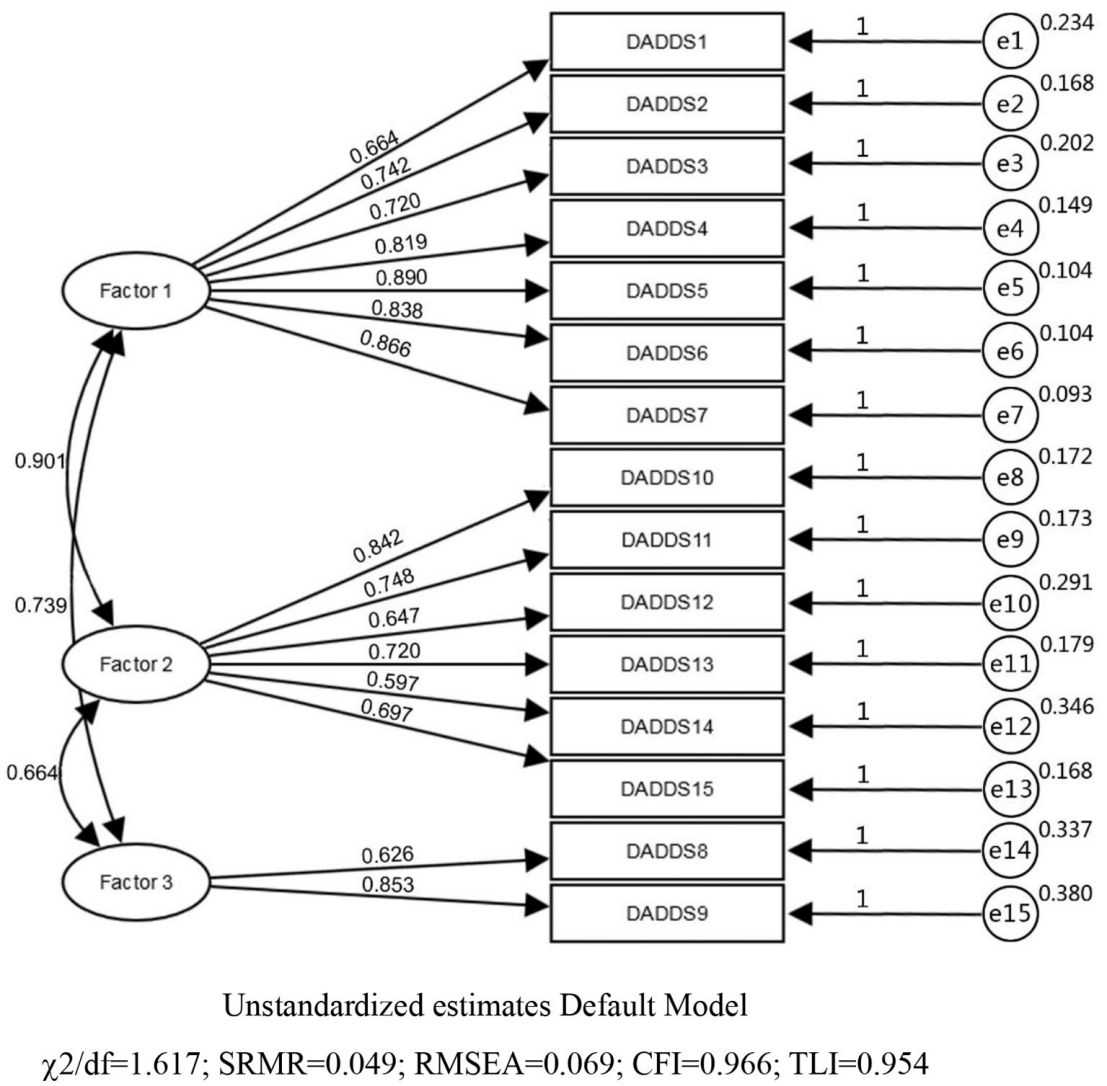
Three factors model in confirmatory factor analysis.

Criterion validity was established as DADDS-C was positively correlated with [PHQ-9 (*r* = 0.602, *p* < 0.001), GAD-7 (*r* = 0.676, *p* < 0.001), subscale of QUAL-EC (*r* = 0.172, *p* = 0.009), subscale of QUAL-EC (*r* = 0.542, *p* < 0.001), and FACIT-sp (*r* = −0.328, *p* < 0.001), subscale of QUAL-EC(*r* = −0.304, *p* < 0.001), subscale of QUAL-EC(*r* = −0.345, *p* < 0.001)]. The three factors-model was analyzed by CFA firstly. Standard loading coefficients of items ranged from 0.597 to 0.890, indicating preferable correlation between items and factors (Figure 1). Acceptable convergent validity was confirmed by Average Variance Extraction (AVE) of 0.502–0.625 and Construct Reliability (CR) of 0.712– 0.906. AVE square root of three factors were: factor 1–0.790, factor 2–0.708, and factor 3–0.748. Correlation coefficients of factors ranged from 0.516 to 0.740. Minimum value of AVE square root (0.708) was smaller but very close to the maximum value of factors correlation coefficient (0.740), indicating acceptable discriminant validity of DADDS-C. χ^2^/df (1.617), SRMR(0.049), RMSEA(0.069), CFI(0.966), TLI (0.954) indicated acceptable overall model fitting validity (Figure 2); two factors-model though had a better discriminant validity, had poorer value than three factors-model with χ^2^/df (3.481), SRMR (0.081), RMSEA (0.140), CFI (0.769), TLI (0.816) (Table 3).

**Table 3:** Comparing Model fits between three factor-model and two factors-model through confirmatory factor analysis.

**Parameter**	**χ^2^**	**df**	**χ^2^/df**	**SRMR**	**RMSEA**	**CFI**	**TLI**
Criteria	–	–	≤ 2.0	<0.08	≤ 0.06	≥0.95	≥0.95
Model fits of the three factors-model	126.157	78	1.617	0.049	0.069	0.966	0.954
Model fits of the two factors-model	309.770	89	3.481	0.081	0.140	0.844	0.816

#### Reliability

Cronbach's alpha for the total scale was 0.938; Omega Hierarchical was 0.880, Omega Total was 0.959. These results showed DADDS-C had great internal consistency reliability.

### Risk Factors for Death Anxiety Among Advanced Cancer Patients

Of the total sample, 43 (16.8%) who scored ≥45 on the DADDS-C was defined positive cases; others were defined as negative cases. Age, education level, medical insurance coverage, PHQ-9 total score, suicide ideation, GAD-7 total score, four subscales for QUAL-EC (symptom burden, relationship with healthcare provider, preparation for end-of-life, life completion), FACIT-sp, which had a significant correlation with the DADDS-C score, were entered into univariate and multivariate Logistics regression analyses for risk factors exploration. Since DADDS-C scores significantly differed by age and educational level, they were also included in the logistics analyses. Multivariate Logistic regression results showed better relationship with health professionals (OR = 0.870, *p* = 0.021) preparation for end of life (OR = 1.225, *p* = 0.008) were associated with less death anxiety (Table 4).

**Table 4:** Results of risk factors for death anxiety from Univariate and Multivariate Logistics Regression Analysis.

**Variables in the Equation**		**B**	**S.E**.	**Wald**	**df**	**Sig**.	**Exp(B)** **(Crude/** **Adjusted OR)**	**95% C.I. for EXP(B)**	
								**Lower**	**Upper**
Step 1^a^	Age	−1.406	0.292	23.251	1	*P* < 0.0001	0.245	0.138	0.434
		−0.706	0.379	3.467	1	0.063	0.494	0.235	1.038
	Education level	0.371	0.136	7.470	1	0.006	1.450	1.111	1.892
		0.358	0.187	3.672	1	0.055	1.431	0.992	2.064
	Medical insurance	0.553	0.339	2.672	1	0.102	1.739	0.896	3.377
		0.684	0.466	2.157	1	0.142	1.983	0.795	4.943
	PHQ9-total score	0.201	0.033	36.327	1	*P* < 0.0001	1.222	1.145	1.305
		0.072	0.063	1.280	1	0.258	1.074	0.949	1.217
	Suicide ideation	0.961	0.211	20.661	1	*P* < 0.0001	2.615	1.728	3.958
		0.084	0.368	0.052	1	0.819	1.088	0.529	2.236
	GAD7-total score	0.231	0.036	40.837	1	*P* < 0.0001	1.260	1.174	1.353
		0.088	0.063	1.923	1	0.166	1.092	0.964	1.236
	Symptom burden-QUAL	0.151	0.059	6.559	1	0.010	1.163	1.036	1.305
		0.036	0.086	0.179	1	0.672	1.037	0.876	1.227
	Relationship with HP-QUAL	−0.153	0.040	14.645	1	*P* < 0.0001	0.858	0.793	0.928
		−0.146	0.060	6.036	1	0.014	0.864	0.769	0.971
	Preparation-for-EOL-QUAL	0.334	0.061	30.507	1	*P* < 0.0001	1.397	1.241	1.573
		0.196	0.074	6.984	1	0.008	1.216	1.052	1.406
	Life-Completion-QUAL	−0.084	0.034	5.902	1	0.015	0.920	0.860	0.984
		0.051	0.061	0.705	1	0.401	1.053	0.934	1.186
	FACIT-sp total score	−0.090	0.022	17.466	1	*P* < 0.0001	0.914	0.876	0.953
		−0.004	0.034	0.013	1	0.908	0.996	0.933	1.064
	Constant	−5.127	2.388	4.611	1	0.032	0.006		

a *Variable(s) entered on step 1: age, education level, medical insurance, PHQ9-total score, Suicide ideation, GAD7-total score, Symptom burden-QUAL, Relationship with HP-QUAL, Preparation for EOL-QUAL, Life Completion-QUAL, FACIT-sp total score*.

## Discussion

The present study demonstrated the validity of the DADDS-C assessing death anxiety in Chinese patients with advanced cancer. The analyses identified three factors on the DADDS-C, two of which correspond to the two factors of Finitude and Dying identified in a recent validation study of the DADDS (29). A third factor of worry about their loved ones was also identified in the present study. Though only two items in the third factor suggesting the potential instability in the subscale of DADDS-C, we have reasons to retain the three factors-model as follow: firstly, CFA result has confirmed this three factors-model was better than two factors-model in our sample; secondly, based on terror management theory, self-esteem containing experiencing sense of meaning and value was positive to protect individuals from death anxiety. Researchers verified that meaning of life, mediated by self-esteem, played positive role on death anxiety in Chinese elderly (40). This is consistent with the finding of Hu et al. that love and fulfilling the family mission were main sources of meaning in life for patients with advanced cancer; being a burden to the beloved ones and causing impact on others, which signified negative influence on family mission, meant loss of meaning of life in Chinese culture (41). Thirdly, the meaning of worrying about beloved ones (item 8 and item 9) was far different from finitude and dying distress. Except for absolutely following strict statistics methods, culture and clinical practice should also be considered to define factors structure. Therefore, we thought this factor should be preserved in DADDS-C for better understanding death anxiety in Chinese advanced cancer patients.

Almost 17% of the participants in this study reported at least moderate death anxiety and these individuals reported a worse relationship with their health care providers and being less prepared for the end of life. Psychological and spiritual concerns are subjective feelings that must be reported by patients themselves. These concerns can be captured in what have been termed patients-reported outcomes (PROs) (42), although distress about dying and death have not been routinely assessed. The present study has demonstrated the validity of the DADDS-C to capture this outcome in our sample. Few well-designed researches on death anxiety was reported among Chinese advanced cancer patients. DADDS-C as a validated instrument would be useful and necessary to facilitate future researches. Discrepancy between our factor structure and western results indicated culture difference to a certain extent. So, using the same instrument world widely used would contribute to further understanding of different cultures' influence on all aspects of death anxiety. Death anxiety was correlated with depression and anxiety, as in other studies (19, 43), but this association was no longer present in logistic regression analyses. Better relationship with healthcare provider and preparation for end-of-life were independent protective factors for death anxiety, suggesting that clinical interventions supporting these factors may be of therapeutic value; researches and clinical management for depression and general anxiety could not fully cover the connotation of death anxiety.

Though conversation about death and dying is often avoided in China (44, 45), the high participant acceptability and 82.5% valid ratio for data collection demonstrated feasibility of utilizing the DADDS-C among Chinese advanced cancer patients. This outcome has been shown to be positively affected by the Managing Cancer and Living Meaningfully (CALM) intervention and will be an important outcome in the randomized controlled trial that we plan to conduct in China.

### Strengths and Limitations

This study is the first validation and application report in China on a special death anxiety measurement designed for patients with advanced cancer and we used relatively rigorous statistical methods and strategy to verify the psychometric properties of DADDS-C. However, limitations deserved further exploration and were collected as follows: (1) Most of the participants were females with breast cancer, in middle age, living with a spouse, having high education, and living in cities. Though we found no association of death anxiety with sex and diagnosis, there should be caution regarding the generalizability of the results. (2) The participants were recruited from ambulatory clinics and inpatients ongoing anti-cancer treatments in our hospital and therefore may not be representative for advanced cancer patients with poor performance status. (3) Only 27 participants completed the DADDS-C a second time 15 days later so that re-test reliability could not be established. (4) Only two items in factor 3 showed potential instability in this subscale; more future studies is needed to focus on this so as to promote general utilization of DADDS-C. (5) This validation study was based on Classic Theory (CTT); Item Response Theory (IRT) was highly recommended by former researchers (46–48) and would be performed in the future research for discriminative value of DADDS-C. (6) Though DADDS-C was obviously a state-like scale as the purpose of developing this scale (capturing death anxiety phenomenon) reported by Lo (28), it was suggested to verify its nature by statistics methods (49). Further researches are needed on the DADDS-C on a more representative sample, with qualitative data regarding the acceptability of the DADDS-C, establishing its re-test reliability, and performing IRT and other statistics methods to get more information and implication about this instrument. There should also be exploration of how demographic and cultural factors may shape the nature and prevalence of death anxiety among Chinese people with advanced cancer.

### Clinical Implications

Three factors-model DADDS-C is practical and preferable to be used for future studies among Chinese patients with advanced cancer. Standard measurement available under our culture background could facilitate more attention to death and dying researches and trigger more focus on this issue in clinical practice for high-quality cancer care. DADDS-C has been used as one of the main outcome measurement for in our RCT of CALM therapy in Chinese patients with advanced cancer. We found better relationship with health professionals and preparation for end of life were associated with less death anxiety, which provided useful information for the interventional study.

## Conclusion

DADDS-C is a practical measurement for death anxiety with good reliability and validity and it could be used in both researches and clinical practice. High prevalence of death anxiety among Chinese patients with advanced cancer reminds medical staff and caregivers associated with cancer care to give more attention to this issue.

## Data Availability Statement

Data supporting findings of this study and supplementary material are available from the corresponding author upon reasonable request.

## Ethics Statement

The studies involving human participants were reviewed and approved by Peking University Cancer Hospital Ethics Committee (No. 2018YJZ24). The patients/participants provided their written informed consent to participate in this study.

## Author Contributions

LT: conceptualization, funding acquisition, investigation, project administration, resources, data curation, methodology, supervision, validation, writing, and review and editing. YZha: data curation, formal analysis, methodology, validation, writing, and reviewing and editing. YP: validation, data curation, methodology, and review and editing. YZho, JL, LS, YH, ZL, and YW: participants recruiting and review. All authors contributed to the article and approved the submitted version.

## Funding

Our study was funded by Beijing Municipal Health and Scientific and Technological Achievements and Appropriate Technology Promotion Projects in China (2018-TG-48, LT, PI).

## Conflict of Interest

The authors declare that the research was conducted in the absence of any commercial or financial relationships that could be construed as a potential conflict of interest.

## Publisher's Note

All claims expressed in this article are solely those of the authors and do not necessarily represent those of their affiliated organizations, or those of the publisher, the editors and the reviewers. Any product that may be evaluated in this article, or claim that may be made by its manufacturer, is not guaranteed or endorsed by the publisher.
